# A Two-Study Comparison of Clinical and MRI Markers of Transition from Mild Cognitive Impairment to Alzheimer's Disease

**DOI:** 10.1155/2012/483469

**Published:** 2012-02-01

**Authors:** D. P. Devanand, Xinhua Liu, Patrick J. Brown, Edward D. Huey, Yaakov Stern, Gregory H. Pelton

**Affiliations:** ^1^Division of Geriatric Psychiatry, New York State Psychiatric Institute, College of Physicians and Surgeons, Columbia University, 1051 Riverside Drive, Unit 126, New York, NY 10032, USA; ^2^Gertrude H. Sergievsky Center and Taub Institute for Research in Alzheimer's Disease and The Aging Brain, Department of Neurology, College of Physicians and Surgeons, Columbia University, 630 West 168th Street, New York, NY 10032, USA; ^3^Department of Biostatistics, Columbia University School of Public Health, 722 West 168th Street, NY 10032, USA

## Abstract

A published predictor model in a single-site cohort study (questionable dementia, QD) that contained episodic verbal memory (SRT total recall), informant report of function (FAQ), and MRI measures was tested using logistic regression and ROC analyses with comparable measures in a second multisite cohort study (Alzheimer's Disease Neuroimaging Initiative, ADNI). There were 126 patients in QD and 282 patients in ADNI with MCI followed for 3 years. Within each sample, the differences in AUCs between the statistical models were very similar. Adding hippocampal and entorhinal cortex volumes to the model containing AVLT/SRT, FAQ, age and MMSE increased the area under the curve (AUC) in ADNI but not QD, with sensitivity increasing by 2% in ADNI and 2% in QD for a fixed specificity of 80%. Conversely, adding episodic verbal memory (SRT/AVLT) and FAQ to the model containing age, Mini Mental State Exam (MMSE), hippocampal and entorhinal cortex volumes increased the AUC in ADNI and QD, with sensitivity increasing by 17% in ADNI and 10% in QD for 80% specificity. The predictor models showed similar differences from each other in both studies, supporting independent validation. MRI hippocampal and entorhinal cortex volumes showed limited added predictive utility to memory and function measures.

## 1. Introduction

Mild cognitive impairment (MCI) often represents a transitional state between normal cognition and Alzheimer's disease (AD) [1, 2]. Accurate prediction of transition from MCI to AD aids in prognosis and targeting early treatment [3]. Episodic verbal memory impairment and informant report of functional deficits in complex social and cognitive tasks are features of incipient AD, and impairment in these domains is associated with transition from MCI to AD [4, 5]. 

Most biomarkers of MCI transition to AD are related to the underlying disease pathology of amyloid plaques and neurofibrillary tangles [6]. Hippocampal and entorhinal cortex atrophy on MRI scan of brain [7], parietotemporal hypometabolism on ^18^FDG PET [8], increased amyloid uptake using PET [9], and decreased amyloid beta-42 (A*β*42) with increased tau/phospho-tau levels in the cerebrospinal fluid (CSF) [10, 11] each significantly predict transition from MCI to AD. The apolipoprotein E *ε*4 allele increases AD risk, but is not a strong biomarker of transition from MCI to AD [3].

 In a meta-analysis, memory deficits appeared to be superior to MRI hippocampal atrophy in predicting transition to AD [12], but studies in the meta-analysis had highly variable subject inclusion/exclusion criteria and assessment methods. There has been a lack of direct head-to-head comparison of clinical and neuroimaging predictors of transition across different studies.

In our single-site study (Questionable Dementia or QD study) that evaluated and followed a broadly defined sample of patients with MCI, a published predictor model that included specific cognitive, functional, olfactory, and MRI measures strongly predicted transition to AD [3]. In the Alzheimer's Disease Neuroimaging Initiative (ADNI) study, cognitive and functional measures and several biomarkers are assessed in samples of MCI, AD, and healthy control subjects at baseline and serially during followup. In this paper, the first goal was to test the accuracy of a combination of predictor variables derived from the QD study to predict transition from MCI to AD in a completely independent ADNI sample. The validation of specific predictor combinations, rather than individual measures, has rarely been done in independent samples. This is essential before specific cut-points, and ranges for specific predictors in such models can be developed with confidence for eventual clinical application. The second goal was to evaluate the relative utility of clinical and MRI measures in predicting transition from MCI to AD.

## 2. Methods

Patients with MCI in the QD and ADNI studies were included, and patients with AD (ADNI) and healthy control subjects (QD and ADNI) were excluded. The 3-year followup samples were chosen because most transitions occur to AD within 3 years of clinical presentation [13].

### 2.1. QD Study

As previously reported, patients 41–85 years old who presented with subjective memory complaints for clinical evaluation to a Memory Disorders Clinic were eligible if they had a Folstein Mini-Mental State Exam (MMSE) score ≥22 out of 30, memory impairment defined as MMSE recall ≤2/3 objects at 5 minutes or a Selective Reminding Test (SRT) delayed recall score >1 SD below norms, and absence of a consensus diagnosis of dementia made by two experienced raters [3]. Patients could also be included if they had other cognitive and functional deficits. This study began before criteria for MCI were published [1, 2]. Baseline MCI subtype using the criterion of >1.5 SD below norms on cognitive tests was determined post hoc by using age, education, and sex-based regression norms derived from 83 healthy control subjects [4]. Using this approach, 73% of patients met the Peterson criteria for single or multidomain amnestic MCI, and this subsample was also compared to ADNI. The presence of specific neurological or major psychiatric disorders led to exclusion [3]. Patients were followed every 6 months for up to 9 years, and the two raters made a consensus diagnosis at each time point. The sample comprised 148 patients with MCI at baseline, and 126 patients were in the 3-year followup sample.

### 2.2. ADNI Study

Data were obtained from the ADNI study (http://adni.loni.ucla.edu/), a project launched in 2003 by the National Institute on Aging, the National Institute of Biomedical Imaging and Bioengineering, the Food and Drug Administration, private pharmaceutical companies, and non-profit organizations as a $60 million, 5-year public-private partnership. The primary goal is to test whether serial magnetic resonance imaging, positron emission tomography, other biological markers, and clinical and neuropsychological assessment can be combined to measure the progression of MCI and early AD.

Participants 55–90 years old were enrolled if they had at least 6 years of education, spoke English or Spanish, agreed to longitudinal followup and neuroimaging tests, had single or multidomain MCI by the Petersen criteria with MMSE scores between 24 and 30, a memory complaint verified by informant, an abnormal memory score (1.5 SD below age-adjusted cutoff) on the Logical Memory II subscale (delayed paragraph recall) from the Wechsler Memory Scale-Revised, and absence of a dementia diagnosis. All participants had a Geriatric Depression Scale score of <6 and a modified Hachinski score of ≤4. For a more detailed account of the inclusion/exclusion criteria, please see http://www.adni-info.org. Raters at each site made consensus diagnoses at six-month intervals that included an evaluation of transition from MCI to AD, which was reviewed by a central committee. Data were obtained from ADNI on October 31, 2010. Of 394 individuals with MCI at baseline evaluation, 282 subjects completed 3 years of followup.

### 2.3. Comparable Baseline Measures Chosen for Analysis from QD and ADNI

In the QD study, the SRT total recall (12 items, 6 trials) was the strongest predictor among the five hypothesized neuropsychological predictors examined [3]. The SRT was not done in ADNI, but the comparable measure of total recall across 6 trials in the Auditory Verbal learning Test (AVLT) was not used for study inclusion criteria and was available. Informant report of the patient's functioning using the Pfeffer Functional Activities Questionnaire (FAQ) total score and MRI hippocampal and entorhinal cortex volumes was additional predictors in the final model in QD [3] that were also assessed in ADNI.

Both studies conducted MRI on 1.5T scanners: a single GE scanner in QD, and GE or Siemens or Philips scanners across 48 sites in ADNI. In QD, hippocampal volume was assessed by a semiautomated method with specific anatomical landmarks used to define hippocampal boundaries, and entorhinal cortex volume was computed from three slices centered at the level of the mammillary bodies [7]. In ADNI, MRI hippocampal and entorhinal cortex volumes were derived from postprocessed image analysis that used FreeSurfer (FS) version 4.3.0 by researchers at the University of California, San Francisco (UCSFFSX); the data are available at http://adni.loni.ucla.edu/. The volume derivation process is described at http://www.loni.ucla.edu/twiki/bin/view/ADNI/ADNIPostProc. For both studies, intracranial volume was a covariate in all analyses of hippocampal and entorhinal cortex volumes. 

### 2.4. Statistical Analyses

Summary statistics were calculated to describe the sample characteristics in the ADNI and QD studies. For each study, Chi-square and *t*-tests were used to detect differences in baseline categorical and continuous variables between MCI patients with and without transition to AD by three years of followup (there were few non-AD dementia cases in both studies). The QD and ADNI studies had different available followup duration times, and therefore survival analysis was not used for comparisons. For both datasets, specific sets of baseline predictors were examined in logistic regression models for the binary outcome of transition to AD within 3 years after baseline evaluation. With each model, sensitivity and specificity were calculated for all possible cut points on the predicted risk of transition to AD to construct receiver operating characteristic (ROC) curves. From the ROC curves, the area under the curve (AUC) was compared statistically between datasets and between nested models within each dataset.

## 3. Results

### 3.1. Demographic and Clinical Features of the Two Samples

Compared to the QD sample, the ADNI sample was older, had a greater proportion of males, had a higher proportion with the apoE *ε*4 allele, and reported greater functional impairment (Table 1). The samples did not differ in years of educational attainment and MMSE scores.

### 3.2. Prediction of Transition from MCI to AD by 3-Year Followup

The majority of patients in ADNI (157/282 or 55.6%) and a minority of patients in QD (33/126 or 26.1%) converted to AD by 3-year followup; the disparity likely related to more stringent inclusion criteria for memory impairment in ADNI compared to QD. Based on logistic regression analyses, the combination of age and MMSE was a poor predictor in ADNI and showed low sensitivity at the fixed level of 90% specificity in QD (top of Table 2). Models that included age with MMSE and specific combinations of AVLT or SRT total recall, FAQ scores, hippocampal and entorhinal cortex volumes showed greater sensitivity, specificity, and predictive accuracy in the QD study compared to ADNI (Table 2).

### 3.3. Comparison of AUCs

**Figure 1:**
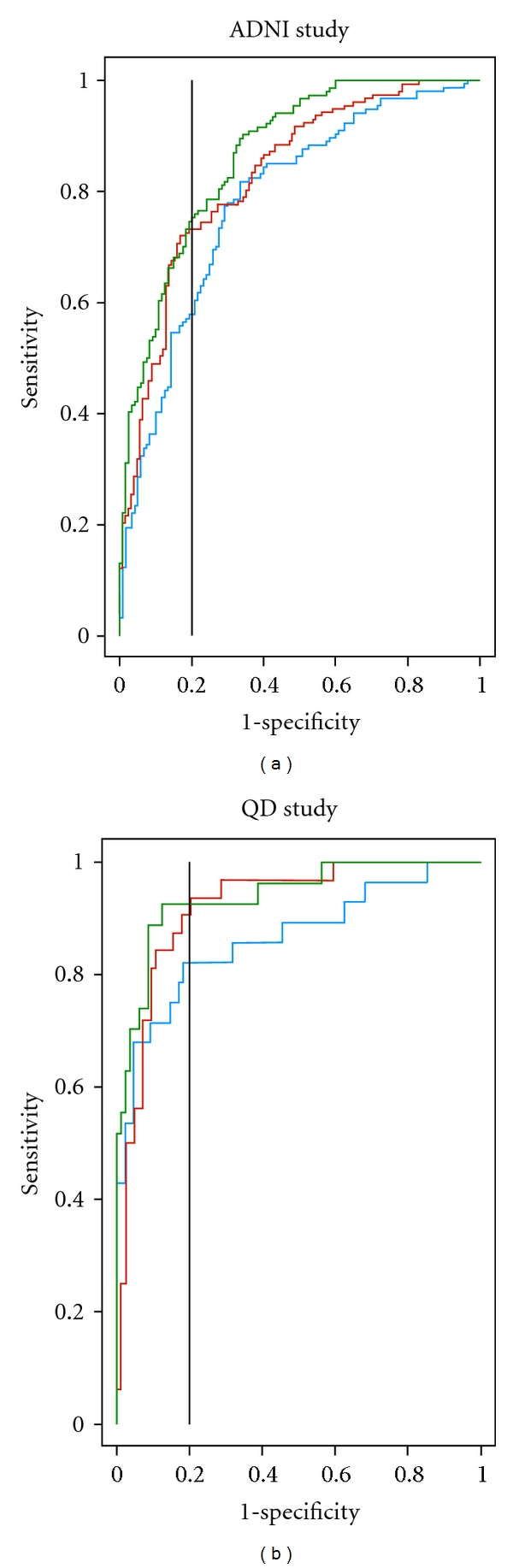
Comparison of receiver operating characteristic (ROC curves) for three statistical models in the ADNI and QD studies. Model 1 (red line) contained age, MMSE, AVLT/SRT and FAQ, Model 2 (blue line) contained age, MMSE, hippocampal and entorhinal cortex volumes, and Model 3 (green line) contained age, MMSE, AVLT/SRT, FAQ, hippocampal and entorhinal cortex volumes. The vertical lines at 80% specificity (0.2 on *x*-axis) indicate 20% false positives.

Three predictor models were compared within and across studies with age and MMSE, which are common clinical indicators, contained in all models. Model 1 included AVLT/SRT and FAQ, Model 2 included hippocampal and entorhinal cortex volumes, and Model 3 included AVLT/SRT, FAQ, and hippocampal and entorhinal cortex volumes (Table 2). In each study, the increase in AUC for Model 1 compared to Model 2 was marginal (around 0.04 in both studies) and not statistically significant (bottom of Table 2). The AUC increased consistently across the two studies when episodic verbal memory (AVLT/SRT) and function (FAQ) measures were added to the model containing the combination of age, MMSE, and hippocampal and entorhinal cortex volumes (*P* < 0.0001 in ADNI and *P* = 0.0254 in QD; Model 2 versus Model 3, bottom of Table 2 and Figure 1), with an appreciable increase in sensitivity for a fixed specificity of 80% and 90% in both ADNI (increases of 17% and 15%, resp.) and QD (increases of 10% and 17%, resp.; top of Table 2 and Figure 1). Conversely, adding hippocampal and entorhinal cortex volumes to AVLT/SRT, FAQ, age, and MMSE significantly increased the AUC in ADNI (*P* = 0.0035) but not in QD (*P* = 0.20) and led to a small increase in sensitivity for a fixed specificity of 80% and 90% in ADNI (increases of 2% and 6%, resp.) and QD (increases of 2% and 7%, respectively, top of Table 2).

In both samples, the differences in AUCs between the three statistical models examined were very similar (bottom of Table 2). Analyses of all combinations of predictors examined are in the supplemental Table  3. (see Supplementary Material available online at doi: 10.1155/2012/483469).

When the QD sample was restricted to patients with baseline amnestic MCI (32/90 transitioned to AD) using comparable criteria to ADNI inclusion criteria for amnestic MCI, the results were similar to the entire QD sample: 80.7% were correctly classified for Model 1, 85.5% for Model 2, and 84.2% for Model 3. AUCs were 0.877 for Model 1, 0.905 in Model 2, and 0.915 in Model 3 without significant differences in AUCs, partly because of reduced sample size. 

## 4. Discussion

Within each sample, QD and ADNI, the differences in AUCs between predictor models were similar, suggesting robustness and generalizability across outpatient settings. When advising patients and families about the likelihood of transition from MCI to AD, a predictor model with specificity over 80% is essential because a false positive rate of over 20% (specificity less than 80%) is clinically unacceptable [14, 15]. In the predictor model, adding hippocampal and entorhinal cortex atrophy to age, MMSE, and the episodic verbal memory and function measures increased sensitivity only to a small extent at fixed specificities of 80% and 90%. These findings suggest limited added utility for MRI hippocampal and entorhinal cortex volumes to clinical assessment of memory and function in predicting transition from MCI to AD. In contrast, adding measures of episodic verbal memory and function to the model that combined age, MMSE, and hippocampal and entorhinal cortex volumes appreciably increased sensitivity for fixed levels of 80% and 90% specificity in both samples. In both studies, the model that included AVLT/SRT, FAQ, and hippocampal and entorhinal cortex volumes with age and MMSE showed the strongest predictive accuracy.

For episodic verbal memory measures, numerical ranges and cutoffs for specific ages and education levels can inform the likelihood of transition to AD. Although delayed recall deficit is typical in AD, both immediate recall (incorporates learning) and delayed recall show comparable predictive accuracy for the transition from MCI to AD [4]. The use of a single episodic memory measure in the predictor models examined does not replace the need for a comprehensive neuropsychological evaluation for diagnostic purposes [4]. Informant reports of FAQ scores reflect instrumental, social, and cognitive functional impairments, but specific cutoffs for prediction of transition to AD are not established [5, 16]. International efforts to standardize MRI imaging parameters and methods of volumetric assessment [17], both of which have varied widely across studies, may lead to the development of specific cutoffs for hippocampal and entorhinal cortex atrophy that improve predictive accuracy.

The use of cognitive markers has some advantages over neuroimaging: objectivity in scoring, comparative economy in expense and time, and reliability. One argument is that episodic verbal memory should not be used as a marker because it is used for inclusion criteria and in the diagnostic process. However, evaluation of severity of episodic verbal memory deficit as a predictor in patients with amnestic MCI who have episodic verbal memory deficits is analogous to the established strategy of evaluating severity of depression as a predictor of clinical course and treatment response in major depression [18]. Further, using memory test scores in prediction creates a statistical handicap, rather than an advantage, by restricting the range in baseline memory test performance [12]. Of note, the AVLT memory measure examined as a predictor in this paper was not part of the study inclusion criteria in ADNI (WMS-R logical memory was used). The same rationale applies to the incorporation of the MMSE, which is widely used and clinically relevant, in predictor analyses even though it is part of the screening criteria for study inclusion.

Informant report of functional impairment using the FAQ was not part of the inclusion criteria in either QD or ADNI, and the definition of MCI by the original Petersen criteria requires the absence of significant functional impairment [1, 2]. Therefore, the use of informant report of functional impairment is independent of the diagnostic criteria for MCI, and our findings indicate that this type of assessment is important in predicting transition to AD [3, 5].

Clinical and neurobiological markers have been incorporated recently into diagnostic classification systems. An international panel used the terms “prodromal dementia” and “predementia” to indicate that neurobiological markers may identify patients with incipient AD who cannot be diagnosed clinically [19]. The new NIA diagnostic criteria separate core clinical criteria from research criteria that employ neurobiological markers [20], partly because diagnostic and predictive accuracy for neurobiological markers has not been fully developed and validated. Our results emphasize the need for such validation.

There have been few comparisons of predictor models between studies. In a comparison of ADNI to a Finnish study, classification performance did not increase after the inclusion of 10 variables that included CSF measures, apolipoprotein E *ε*4, MRI measures, age, and education [21]. The overall model was not strong, possibly because key cognitive and functional measures were excluded. Another study compared different samples of patients with MCI who had ^18^FDG PET with generally positive results [22] but without cut-points for clinical application. Our report represents a novel independent validation of predictor models that included clinical, memory, functional, and MRI measures. The consistency in the differences between models in each study indicates that this two-study comparison is broader and more clinically relevant than prior validation attempts [21, 22].

From the ADNI database, several reports show moderate predictive accuracy for weighted scores within a global cognitive test [23] and moderately strong predictive accuracy for specific neuropsychological test scores [24], consistent with other studies [4]. The best possible fit from a high-dimensional pattern classification approach using ADNI MRI data [25] led to results similar to our report that used volumetric measures, but other MRI analytic strategies using ADNI data have led to lower predictive accuracy [26, 27]. Entorhinal cortex volume enhanced prediction in both ADNI and QD in our comparisons, supporting the evaluation of entorhinal cortex volume as a predictor [7].

There were some limitations to this paper. The two samples differed in sex and age distribution and cognitive test scores, significant episodic verbal memory deficits were required in ADNI compared to broader inclusion criteria in QD that may partly account for higher transition rates in ADNI, and different episodic verbal memory measures and different MRI volumetric assessment methods were compared. Nonetheless, within each sample for several combinations of predictors the differences in AUCs were similar. The high transition rate in ADNI suggests that some patients diagnosed with MCI by 3-year followup may convert in subsequent years, likely leading to a higher rate of false negatives in ADNI. This may partly explain the lower accuracy for predictor combinations in ADNI. In ADNI, the smaller number of patients at 3-year followup was partly related to some recently recruited patients not yet having had the opportunity to reach 3-year followup at the time of data analysis for this paper. This issue also precluded the use of survival analysis in this sample. In QD, we derived the strongest predictors from a set of a priori measures in a large neuropsychological test battery and examined comparable measures from the shorter ADNI neuropsychological assessment. While administering a comprehensive neuropsychological test battery is important for diagnostic purposes, our clinically relevant approach of examining individual measures facilitates comparison across studies and demonstrates the predictive strength of even a single episodic verbal memory test. Baseline MRI measures were examined because serial MRI measures were not available in QD. It remains unclear if serial imaging measures are superior to baseline imaging in predicting long-term outcome [28]. Serial imaging measures provide useful information about structural changes associated with disease progression, but they are expensive, not current clinical practice, and not useful in early converters. Cerebrovascular disease may contribute to cognitive decline in these patients [19, 20]. However, hyperintensities, lacunes, and infarcts could not be assessed systematically in QD because of the MRI sequences obtained (no FLAIR or comparable sequence) and therefore could not be compared with ADNI. Absent neuropathological validation, we considered examining CSF measures from ADNI (not done in QD) for in vivo validation of transition to AD, but CSF was not collected in approximately half the ADNI sample and neuropathological validation of CSF tau and A*β* abnormalities has not been established.

In QD, the pathophysiological measure [19] of olfactory identification deficits (not done in ADNI) strongly predicted transition to AD with limited overlap in prediction with the SRT and MRI measures [3, 29]. In ADNI, ^18^FDG indices (not done in QD) significantly predicted transition to AD and were superior to the ADAS-cog [8], but the ADAS-cog is a global cognitive measure used primarily in clinical trials of AD patients and is not established as a strong predictor of transition from MCI to AD. PET amyloid imaging discriminates among AD, MCI, and controls [30] and correlates at autopsy with amyloid plaques [9]. However, approximately 10–30% of healthy controls show increased amyloid uptake [30] and whether these subjects have incipient AD needs confirmation in long-term followup studies. The sensitivity and specificity of CSF levels of A*β*42 and tau/phospho tau, and their ratio, for predicting MCI transition to AD in ADNI [31] and in a European multicenter study [32] ranged from 65% to 75%, which is slightly lower than that in other reports [10, 11]. For CSF markers, further refinement of assay technique and validation in long-term followup studies are needed to establish more definitive cut-points for individual and ratio measures that have varied to some extent across studies [10, 11, 32].

This report suggests that volumetric evaluation of medial temporal lobe atrophy adds only marginally to the information obtained by cognitive testing and assessment of episodic memory, and it cannot yet be recommended for wide clinical use to assess the risk of patients with MCI being diagnosed with AD during followup. In the clinic, visual inspection ratings are likely to lead to lower predictive accuracy than either the QD or ADNI volumetric assessments. Structural neuroimaging with MRI remains useful to rule out specific causes of cognitive impairment, for example, stroke, tumor. A key conclusion from this report is that conducting neuropsychological evaluation is important, and interviewing family members or other informants about the patient's functioning may be at least as important as conducting an MRI scan. Several clinical and neurobiological markers, including cognitive test scores, functional ability, and MRI and ^18^FDG PET measures, are influenced considerably by age and other demographic factors, and their utility needs to be evaluated in more heterogeneous samples. The comparative predictive utility of clinical and neurobiological markers needs further assessment across different populations as these measures improve in predictive accuracy.

## Supplementary Material

Table 3. Predictive accuracy for classification of transition to Alzheimer's Disease (AD) by 3 years of follow-up, using a threshold of 0.5 on predicted risk derived from the logistic regression models. Area under the curve was derived from Receiver Operating Characteristic (ROC) analyses.

## Figures and Tables

**Table 1:** Baseline sample characteristics of patients with MCI with three years of followup.

Variables	Alzheimer's Disease Neuroimaging Initiative (ADNI)				Questionable Dementia (QD) study			

	Total *N* = 282 %	Nonconverter *N* = 125 %	Converter *N* = 157 %	Group difference Chi-square *P* value	Total (*N* = 126) %	Not converted (*N* = 93) %	Converted to AD (*N* = 33) %	Group difference Chi-square *P* value
Gender (% Male)	67.02	76.0	59.87	0.0063	26.19	48.39	39.39	0.3154
Race (%)								
Caucasian	92.20	92.00	92.36	0.8913	75.40	77.42	69.70	0.8452
Hispanic	2.84	2.40	3.18		16.67	15.05	21.21	
African American	2.48	2.40	2.55		5.56	5.38	6.06	
Other	2.48	3.20	1.91		2.40	2.15	3.03	
ApoE *ε*4	56.03	40.00	68.35	<0.0001	27.27	25.27	33.33	0.5332

	Mean (SD)	Mean (SD)	Mean (SD)	*t*-test	Mean (SD)	Mean (SD)	Mean (SD)	*t*-test

Age (years)	74.59 (7.30)	74.63 (7.66)	74.56 (7.03)	0.9351	67.31 (9.72)	65.24 (9.69)	73.12 (7.21)	<0.0001
Education (years)	15.76 (2.88)	15.95 (2.81)	15.60 (2.94)	0.3074	15.18 (4.03)	15.58 (3.77)	14.06 (4.58)	0.0507
MMSE	27.08 (1.80)	27.62 (1.74)	26.66 (1.73)	<0.0001	27.52 (2.21)	28.02 (1.99)	26.12 (2.20)	<0.0001
AVLT/SRT	34.14 (10.90)	39.73 (12.16)	29.69 (7.16)	<0.0001	42.66 (9.49)	45.60 (8.26)	34.09 (7.50)	<0.0001
FAQ	2.84 (2.79)	1.61 (2.08)	3.82 (2.90)	<0.0001	1.69 (2.05)	1.28 (1.84)	2.73 (2.21)	0.0002

	*N* = 274	*N* = 120	*N* = 154	*t*-test	*N* = 118	*N* = 89	*N* = 29	*t*-test

Hippocampal volume	6.30 (1.10)	6.78 (1.01)	5.92 (1.02)	<0.0001	4.20 (0.73)	4.37 (0.62)	3.69 (0.74)	<0.0001
Entorhinal cortex volume	0.33 (0.08)	0.36 (0.07)	0.30 (0.07)	<0.0001	0.45 (0.10)	0.47 (0.09)	0.38 (0.09)	<0.0001
Intracranial volume	1580.79 (166.15)	1602.50 (152.48)	1563.69 (174.67)	0.0549	1306.60 (126.83)	1317.43 (128.55)	1273.71 (117.49)	0.0919

MMSE: Mini-Mental State Exam, AVLT: Auditory Verbal Learning Test (sum of 6 trials), SRT: Selective Reminding Task (sum of 6 trials), FAQ: Pfeffer's Functional Activities Questionnaire (10 items), SD: standard deviation, AD: Alzheimer's disease. Hippocampal, entorhinal, and intracranial volumes are in cubic centimeters. Entorhinal cortex volumes measured in cubic millimeters in ADNI were converted to cubic centimeters. Intracranial volume in ADNI covered all intracranial structures including the cerebellum, but intracranial volume in QD was restricted to supratentorial intracranial volume.

**Table 2:** Predictive accuracy of specific combinations of predictor variables for classification of transition to Alzheimer's disease (AD) by 3 years of followup in two independent samples (ADNI and QD) of older adults with Mild Cognitive Impairment, and comparisons of three predictor models.

		ADNI			QD		
Model	Predictor variables	AUC (SE)	Sensitivity at specificity = 80% (90%)	Correct classification %	AUC (SE)	Sensitivity at specificity = 80% (90%)	Correct classification %
	Age	0.497	12.74 (5.73)	55.67	0.739	52.61 (29.85)	73.02
	MMSE	0.655	37.88 (19.20)	65.54	0.778	41.41 (26.79)	76.00
	Hippocampal vol.	0.725	48.05 (34.42)	64.60	0.753	62.07 (41.38)	80.51
	Entorhinal volume	0.718	50.65 (35.71)	67.16	0.773	67.86 (50.00)	80.34
	AVLT	0.756	49.47 (25.16)	44.33	0.849	71.63 (53.13)	80.00
	FAQ	0.738	49.05 (35.90)	44.33	0.708	45.46 (32.83)	75.42
	Age, MMSE	0.659	36.94 (18.79)	63.48	0.821	72.73 (39.39)	76.00
	Hippocampal and entorhinal volumes	0.744	55.84 (35.71)	68.98	0.824	67.86 (67.86)	88.03
	AVLT/SRT and FAQ	0.811	62.74 (42.68)	72.70	0.879	78.13 (59.38)	82.05
Model 1	Age, MMSE, AVLT/SRT and FAQ	0.828 (0.024)	73.25 (49.05)	73.40	0.921 (0.027)	90.63 (81.25)	87.07
Model 2	Age, MMSE, Hippocampal and entorhinal volumes	0.783 (0.028)	57.79 (40.26)	73.72	0.866 (0.046)	82.14 (71.43)	87.07
Model 3	Age, MMSE, AVLT/SRT, FAQ, Hippocampal and entorhinal volumes	0.865 (0.022)	75.33 (55.20)	77.01	0.940 (0.027)	92.59 (88.89)	89.72

Model comparisons		AUC difference	*P* value		AUC difference	*P* value	

Model 1 versus Model 2		0.0396	0.2271		0.0428	0.3618	
Model 1 versus Model 3		0.0428	0.0035**		0.0282	0.1979	
Model 2 versus Model 3		0.0824	0.0001**		0.0710	0.0254*	

A threshold of 0.5 was used on predicted risk derived from the logistic regression models. Area under the curve (AUC) was derived from receiver operating characteristic (ROC) analyses. *N* = 282 (157 converters) in ADNI and *N* = 126 (33 converters) in QD. The differences between models in AUCs are slightly different from the direct subtraction of AUCs between models because of missing data that ranged from 1% to 4% for the variables examined in ADNI and 1% to 5% for the variables examined in QD.

*P < 0.05, **P < 0.01.
