# Supplementary material for: A Two-Study Comparison of Clinical and MRI Markers of Transition from Mild Cognitive Impairment to Alzheimer's Disease
Source: Int J Alzheimers Dis. 2012 Feb 1;2012:483469. doi: 10.1155/2012/483469 (PMC3296186; doi:10.1155/2012/483469)
Supplement: Supplementary file 1 — Table 3. Predictive accuracy for classification of transition to Alzheimer's Disease (AD) by 3 years of follow-up, using a threshold of 0.5 on predicted risk derived from the logistic regression models. Area under the curve was derived from Receiver Operating Characteristic (ROC) analyses. [file 483469.f1.docx]

Table 3. Predictive accuracy for classification of transition to Alzheimer’s Disease (AD) by 3 years of follow-up, using a threshold of 0.5 on predicted risk derived from the logistic regression models. Area under the curve was derived from Receiver Operating Characteristic (ROC) analyses.

| Predictor  Variables | N: converters to AD / total | Combined predictors  p-value | AUC | Sensitivity at  Specificity =  80% (90%) | Correct classification  % | Positive predictive value  % | Negative predictive value  % |
| --- | --- | --- | --- | --- | --- | --- | --- |
| **Alzheimer’s Disease Neuroimaging Initiative (ADNI)** | | | | | | | |
| Age | 157 / 282 | .9348 | 0.497 | 12.74 (5.73) | 55.67 | 55.67 | NA |
| MMSE | 157 / 282 | <.0001 | 0.655 | 37.88 (19.20) | 65.54 | 67.70 | 60.33 |
| Hippocampal Vol. | 154 / 274 | <.0001 | 0.725 | 48.05 (34.42) | 64.60 | 66.29 | 61.62 |
| Entorhinal Volume | 154 / 274 | <.0001 | 0.718 | 50.65 (35.71) | 67.16 | 69.05 | 64.15 |
| AVLT | 157 / 282 | <.0001 | 0.756 | 49.47 (25.16) | 44.33 | NA | 44.33 |
| FAQ | 157 / 282 | <.0001 | 0.738 | 49.05 (35.90) | 44.33 | NA | 44.33 |
| Age, MMSE | 157 / 282 | .5446  <.0001 | 0.659 | 36.94  (18.79) | 63.48 | 65.52 | 60.19 |
| Hippocampal and Entorhinal Volumes | 154 / 274 | .0005  .0087 | 0.744 | 55.84  (35.71) | 68.98 | 70.41 | 66.67 |
| AVLT, FAQ | 157 / 282 | <.0001  <.0001 | 0.811 | 62.74  (42.68) | 72.70 | 74.10 | 70.69 |
| Age, MMSE, Hippocampal and Entorhinal Volumes | 154 / 274 | .0011  .0028  <.0001  .0187 | 0.783 | 57.79  (40.26) | 73.72 | 73.84 | 73.53 |
| Age, MMSE,  AVLT, FAQ | 157 / 282 | .2194  .0030  <.0001  <.0001 | 0.828 | 73.25  (49.05) | 73.40 | 75.31 | 70.83 |
| AVLT, FAQ,  Hippocampal and Entorhinal Volumes | 154 / 274 | <.0001  <.0001  .0137  .0146 | 0.848 | 73.38  (46.10) | 77.74 | 79.62 | 75.21 |
| Age, MMSE, AVLT, FAQ,  Hippocampal and Entorhinal Volumes | 154 / 274 | .0057  .0467  <.0001  <.0001  .0027  .0305 | 0.866 | 75.33  (55.20) | 77.01 | 77.91 | 75.68 |
| **Questionable Dementia (QD) study** | | | | | | | |
| Age | 33 / 126 | .0002 | 0.739 | 52.61 (29.85) | 73.02 | 46.67 | 76.58 |
| MMSE | 33 / 125 | .0001 | 0.778 | 41.41 (26.79) | 76.00 | 61.54 | 77.68 |
| Hippocampal Vol. | 29 / 118 | <.0001 | .753 | 62.07 (41.38) | 80.51 | 75.00 | 81.13 |
| Entorhinal Volume | 28 / 117 | <.0001 | .773 | 67.86 (50.00) | 80.34 | 72.73 | 81.13 |
| SRT | 32 / 125 | <.0001 | .849 | 71.63 (53.13) | 80.00 | 66.67 | 82.69 |
| FAQ | 33 / 118 | .0012 | .708 | 45.46 (32.83) | 75.42 | 62.50 | 77.45 |
| Age, MMSE | 33 / 125 | .0006  .0005 | 0.821 | 72.73  (39.39) | 76.00 | 56.52 | 80.39 |
| Hippocampal and Entorhinal Volumes | 28 / 117 | .0002  .0001 | 0.824 | 67.86  (67.86) | 88.03 | 88.89 | 87.88 |
| SRT, FAQ | 32 / 117 | <.0001  .0056 | 0.879 | 78.13  (59.38) | 82.05 | 70.37 | 85.56 |
| Age, MMSE, Hippocampal and Entorhinal Volumes | 28 / 116 | .2823  .0150  .0184  .0008 | 0.866 | 82.14  (71.43) | 87.07 | 88.24 | 86.87 |
| Age, MMSE, SRT, FAQ | 32 / 116 | .0065  .0830  .0007  .0055 | 0.921 | 90.63  (81.25) | 87.07 | 79.31 | 89.66 |
| SRT, FAQ,  Hippocampal and Entorhinal Volumes | 27 / 108 | .0005  .0382  .0039  .0023 | 0.932 | 92.59  (77.78) | 90.09 | 85.19 | 91.67 |
| Age, MMSE, SRT, FAQ,  Hippocampal and Entorhinal Volumes | 27 / 107 | .4543  .5241  .0077  .0245  .0384  .0047 | 0.940 | 92.59  (88.89) | 89.72 | 86.36 | 90.59 |

MMSE = Mini Mental State Exam, AVLT = Auditory Verbal Learning Test (total of 6 learning trials), SRT = Selective Reminding Task (sum of 6 trials), FAQ = Pfeffer Functional Activities Questionnaire (informant report), AD = Alzheimer’s disease.
